# A Novel Sensing Method to Detect Malachite Green Contaminant on Silicon Substrate Using Nonlinear Optics

**DOI:** 10.3390/mi15101227

**Published:** 2024-09-30

**Authors:** Muhammad Ahyad, Hendradi Hardhienata, Eddwi Hesky Hasdeo, Sasfan Arman Wella, Faridah Handayasari, Husin Alatas, Muhammad Danang Birowosuto

**Affiliations:** 1 Theoretical Physics Division, Department of Physics, IPB University, Meranti Avenue, Wing S Building, Dramaga Campus of IPB, Bogor 16680, West Java, Indonesia; muhammadahyad@apps.ipb.ac.id (M.A.); alatas@apps.ipb.ac.id (H.A.); 2 Research Center for Quantum Physics, National Research and Innovation Agency (BRIN), South Tangerang 15314, Banten, Indonesia; eddwi.hesky.hasdeo@brin.go.id (E.H.H.); sasfan.arman.wella@brin.go.id (S.A.W.); 3 Department of Physics and Materials Science, University of Luxembourg, 1511 Luxembourg, Luxembourg; 4 Department of Food Technology, Faculty of Halal Food Science, Djuanda University, Jl. Tol Ciawi No.1, P.O. Box 35, Bogor 16720, West Java, Indonesia; faridah.handayasari@unida.ac.id; 5 Łukasiewicz Research Network—PORT Polish Center for Technology Development, Stabłowicka 147, 54-066 Wrocław, Poland; muhammad.birowosuto@port.lukasiewicz.gov.pl

**Keywords:** nonlinear optics, malachite green (MG), second-harmonic generation (SHG), simplified bond hyperpolarizability model (SBHM)

## Abstract

We propose a nonlinear-optics-based nanosensor to detect malachite green (MG) contaminants on semiconductor interfaces such as silicon (Si). Applying the simplified bond hyperpolarizability model (SBHM), we simplified the second-harmonic generation (SHG) analysis of an MG-Si(111) surface and were able to validate our model by reproducing experimental rotational anisotropy (RA) SHG experiments. For the first time, density functional theory (DFT) calculations using ultrasoft pseudopotentials were implemented to obtain the molecular configuration and bond vector orientation required by the SBHM to investigate and predict the second-harmonic generation contribution for an MG-Si 001 surface. We show that the SBHM model significantly reduces the number of independent components in the nonlinear tensor of the MG-Si(111) interface, opening up the possibility for real-time and non-destructive contaminant detection at the nanoscale. In addition, we derive an explicit formula for the SHG far field, demonstrating its applicability for various input polarization angles. Finally, an RASHG signal can be enhanced through a simulated photonic crystal cavity up to 4000 times for more sensitivity of detection. Our work can stimulate more exploration using nonlinear optical methods to detect and analyze surface-bound contaminants, which is beneficial for environmental monitoring, especially for mitigating pollution from textile dyes, and underscores the role of nonlinear optics in real-time ambient-condition applications.

## 1. Introduction

Environmental pollution remains a critical challenge for both human society and other living organisms, driven by factors such as population growth, industrial activities, and urbanization. One of the major contributors to this pollution is the improper disposal of dyes, particularly from textile industry wastewater [1]. Among these pollutants, organic dyes such as malachite green (MG) are particularly concerning, as noted in Ref. [2]. Malachite green is extensively utilized across various industries, including textiles, oil refining, paper production, healthcare, and aquaculture [2]. However, its presence in wastewater poses significant environmental risks due to its toxicity and potential to harm ecosystems.

Studies, such as those by Sudova et al. (2007) [3], have demonstrated that MG exhibits carcinogenic, mutagenic, and teratogenic effects in mammals, further emphasizing its hazardous nature. Therefore, there is a pressing need to develop effective methods for detecting these contaminants, particularly those that may interact with MG, to mitigate their impact on the environment. Although malachite green (MG) is prohibited for use in food-producing animals within the European Union (EU), its presence has been detected in aquaculture products, indicating its illicit use and underscoring the need for enhanced regulatory oversight. The European Food Safety Authority (EFSA) has established an action threshold of 2 μg/kg for MG and its metabolite, leucomalachite green (LMG), yet the recent recommendations advocate for a lower safety threshold to provide better protection [4]. The data collected by the EU between 2002 and 2014 revealed significant non-compliance, with 548 aquaculture samples failing to meet the existing standards, suggesting that a reassessment of the current safety limits is crucial to protect public health [4].

Various methodologies have been employed to analyze organic pollutants, including the use of scanning electron microscopy (SEM) and transmission electron microscopy (TEM), as demonstrated in Moustafa’s 2023 study [5]. These techniques enable the observation of material morphology and support the interpretation of FTIR results to evaluate molecular bonding groups. Such characterization techniques provide valuable insights into both the material’s structure and the nature of its bonding groups. However, SEM analysis requires conductive samples, presenting difficulties when studying non-conductive organic pollutants like malachite green (MG). Additionally, the extensive sample preparation needed to avoid damage limits the use of SEM and TEM for real-time visualization of dynamic processes. The traditional methods for detecting and analyzing organic pollutants often depend on chromatographic techniques, such as gas chromatography (GC) and liquid chromatography (LC), which are not only time-intensive but also require large sample volumes [6,7,8].

Nonlinear optics offers a unique approach to studying organic pollutants by leveraging the property that certain materials exhibit nonlinear optical behavior under high-intensity illumination. In the context of organic pollutants, nonlinear optics enables the examination of interactions between light and pollutants, revealing valuable insights into their molecular composition, physical characteristics, and environmental behavior. A key advantage of nonlinear optics is its ability to deliver real-time data on the environmental dynamics of organic pollutants. This is facilitated by fiber optics and other sensing technologies that can be deployed in situ to monitor the distribution and presence of pollutants in their natural settings. Beyond its analytical strengths, nonlinear optics provides practical advantages for the detection of organic pollutants, particularly at low concentrations.

**Figure 1:**
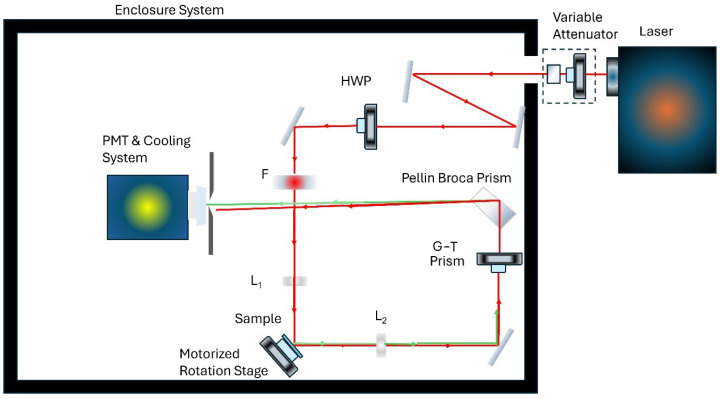
RASHG experimental setup to sense the MG contaminant through its SHG intensity profile. Figure recreated from Ref. [7]. (Abbreviations: HWP: half-wave plate; F: filter; L${‌}_{1}$: objective lens, L${‌}_{2}$: collective lens; G–T: Glan–Materials polarizing prism; PMT: photomultiplier).

Enhanced optical detection techniques, such as second-harmonic generation (SHG) and four-wave mixing (FWM), enable the highly sensitive and precise identification of organic pollutants in water and soil samples [9,10]. For instance, the studies in Refs. [10,11] explored the rotational anisotropy of SHG (RASHG) at the MG–silica interface and the solid–liquid interface, respectively. Figure 1 presents an example of an RASHG setup based on Ref. [12]. This study is based on the analysis of experimental data using theoretical concepts from nonlinear optics, specifically by examining the role of the nonlinear optical susceptibility of the interface materials, silica and malachite green (MG). There are varying interpretations of the nonlinear contributions to the experimental data, and these often lead to contentious debates. For instance, Kolthammer et al. (2005) [12] suggested that the second-harmonic generation (SHG) response may stem from surface and bulk quadrupoles or the magnetic dipole effect. It was here that the simplified bond hyperpolarizability model (SBHM) was first developed and applied to prove that the contribution arises mainly from the surface by the work in Ref. [13].

Afterwards, Hardhienata et al. (2021) [14] developed the SBHM further to explore the SHG signal contributions from quadrupole and spatial dispersion showing that bulk effects must also be considered. Therefore, both surface and bulk SHG sources plays a role in the total SHG intensity in Si. Moreover, the SBHM model demonstrates broad applicability in the field of nonlinear optics, particularly for surface kinetics and structural investigations at the nanoscale. This model can be effectively applied when the primary assumption holds—that dipoles oscillate anharmonically along the bond orientation. Under this condition, the SBHM model has been successfully employed to investigate highly symmetrical semiconductor surfaces, such as Si [7,13,15] and ZnO [16].

The SBHM model can also be utilized to analyze the impact of external factors such as temperature and humidity, particularly in studying phase transitions or structural changes due to temperature variations. For instance, recent research on perovskites [17,18] demonstrated the model’s ability to distinguish between two phases by comparing second-harmonic generation (SHG) intensity results with experimental data. In these instances, the bond orientation of each phase is integrated into the model and validated through experimentation. Similarly, the SBHM model can be adapted to examine changes in the molecular orientation at surfaces resulting from shifts in humidity, applying the same methodology used in temperature-dependent investigations.

In this work, we apply a theoretical and modeling approach where we aim to detect the presence of malachite green (MG) pollutants under room conditions. While it is indeed interesting to consider temperature and humidity changes, it is currently beyond the scope of this work. However, once rotational anisotropy SHG data for various temperatures and humidity levels are available, the SBHM model can certainly be applied to determine whether there are changes in the surface molecular orientation. From a theoretical point of view, changes in temperature will excite the vibrational modes (phonons) in the molecule, which may alter the symmetry and consequently affect the SHG intensity. The nonlinear optics method through the SBHM approach is used to investigate MG as an organic contaminant on the Si(111) surface. We also investigate for the first time the adsorption geometry of the MG molecule on the Si(001) surface by means of density functional theory (DFT) calculations using ultrasoft pseudopotentials, as implemented in the Quantum ESPRESSO [19]. The exchange–correlation interaction was described by the generalized gradient approximation (GGA) proposed by Perdew–Burke–Ernzerhof [20]. The integration of DFT to obtain the optimal structure for the SBHM bond vector has never been performed in previous work. Not to mention, the application of a simulated photonic crystal device (PCD) to improve RASHG has never been explored previously.

## 2. Materials and Methods

Nonlinear optics investigates the phenomena that arise from changes in the optical properties of materials as a result of their interaction with light. Notably, it is only laser light of sufficiently high intensity that can induce such alterations in a material’s optical characteristics. When exposed to an external field of high intensity, a dipole moment is generated within a specific region of the material, a process known as polarization. The total polarization can be expressed as follows:

(1) $$P_{i}=\varepsilon_{0}\left(\chi_{ij}^{\left(1\right)}E_{j}+\chi_{ijk}^{\left(2\right)}E_{j}E_{k}+\chi_{ijkl}^{\left(3\right)}E_{j}E_{k}E_{l}+\cdots \right).$$

here, $\varepsilon_{0}$ represents the vacuum permittivity, $\chi^{\left(1\right)}$ denotes the linear susceptibility, and $\chi^{\left(2\right)}$ and $\chi^{\left(3\right)}$ refer to the nonlinear susceptibilities. The electric fields $E_{j},E_{k}$, and $E_{l}$ correspond to the input fields, each oriented in specific directions. In this study, the Cartesian coordinates *x*, *y*, and *z* are adjusted in accordance with the material’s symmetry. The material’s response to an external electric field, which induces polarization, can be explained by its susceptibility. In the case of second-order polarization, related to second-harmonic generation, the susceptibility tensor can be expressed as follows:

(2) $$\chi_{ijk}^{\left(2\right)}=\left(\begin{array}{c} \left(\begin{array}{ccc} \chi_{111} & \chi_{121} & \chi_{131}\\ \chi_{112} & \chi_{122} & \chi_{132}\\ \chi_{113} & \chi_{123} & \chi_{133} \end{array}\right)\\ \left(\begin{array}{ccc} \chi_{211} & \chi_{221} & \chi_{231}\\ \chi_{212} & \chi_{222} & \chi_{232}\\ \chi_{213} & \chi_{223} & \chi_{233} \end{array}\right)\\ \left(\begin{array}{ccc} \chi_{311} & \chi_{321} & \chi_{331}\\ \chi_{312} & \chi_{322} & \chi_{332}\\ \chi_{313} & \chi_{323} & \chi_{333} \end{array}\right) \end{array}\right).$$

As indicated in Equation (2), the tensor $\chi_{ijk}^{\left(2\right)}$ comprises 27 components. According to Einstein’s notation, the indices i,j, and *k* denote a three-dimensional spatial system, which can be represented in Cartesian coordinates along the *x*-, *y*-, and *z*-axes, corresponding to the molecule’s symmetry. The number of independent components of the tensor can be reduced due to the molecule’s symmetry. For instance, in non-resonant effects, Kleinmann symmetry must be observed, and geometric symmetry can further limit the number of independent tensor components [14,21]. For a molecule exhibiting $C_{2v}$ symmetry, only a limited number of non-zero components are present in the susceptibility tensor, specifically $\chi_{xyz}$ and $\chi_{xzy}$.

**Figure 2:**
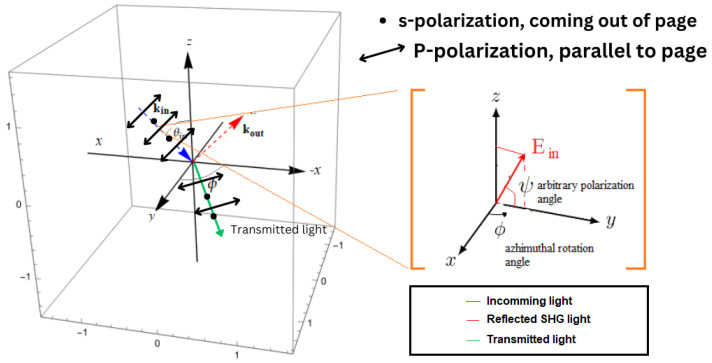
Illustration of experimental configuration and applied coordinate system.

The applied coordinate system in our model is presented in Figure 2. The incoming fundamental and outgoing SHG fields are propagating along the xz plane. In this work, we do not limit ourselves to the four standard incoming *p*- and *s*-polarized incoming fields but instead generalized the model for arbitrary input polarization:

(3) $$E_{\mathrm{loc}}=\left(\begin{array}{c} \cos\theta_{i}\sin\psi \\ \cos\psi \\ \sin\theta_{i}\sin\psi \end{array}\right)$$

where $\theta_{i}$ is the incoming light angle relative to the *z*-plane and ψ is the arbitrary polarization angle, which we will analyze in this work by a regular interval of 10${‌}^{\circ }$. From the yellow inset in Figure 2, it is obvious that, when $\psi =0^{\circ }$, we have a fully *s*-polarized incoming field, and, when $\psi =90^{\circ }$, we have a fully *p*-polarized incoming field.

As proposed by Powell [13] and Aspnes [22] regarding the SBHM method, the model is built based on the assumption that the nonlinear radiation is generated by anharmonic oscillations by only the dipole along the atomic bond direction. This simple model has successfully described the azimuthal-angle-dependent SHG intensity for Si(111) and Si(001) [7,14]. In SBHM, the second-order polarization is formulated as

(4) $$P^{\left(2\right)}=\frac{1}{V}\sum_{i}{\left(\alpha_{2i}\left(R^{\left(z\right)}\left(\varphi \right)\cdot {\hat{b}}_{i}\right)⊗\left(R^{\left(z\right)}\left(\varphi \right)\cdot {\hat{b}}_{i}\right)⊗\left(R^{\left(z\right)}\left(\varphi \right)\cdot {\hat{b}}_{i}\right)E_{\mathrm{in}}\right)}^{2}$$

(5) $$R^{\left(z\right)}\left(\varphi \right)=\left[\begin{array}{ccc} \cos\varphi & -\sin\varphi & 0\\ \sin\varphi & \cos\varphi & 0\\ 0 & 0 & 1 \end{array}\right]$$

where $P_{D}^{2}$ is the polarization occurring at the surface section and $E_{\mathrm{in}}$ is the input electric field, while $\alpha_{2}$ is the hyperpolarizability for 2nd-order nonlinear polarization. The $R^{\left(z\right)}\left(\varphi \right)$ is the rotation matrix about the *z*-axis, and $b_{i}$ is the vector of each bond. The form of the rotation matrix $R^{\left(z\right)}\left(\varphi \right)$ defines the rotation matrix about the *z*-axis, which can be written in the form of Equation (5) [7].The bond vector $b_{i}$ indicates the dipole orientation of individual bonds silicon and MG.

**Figure 3:**
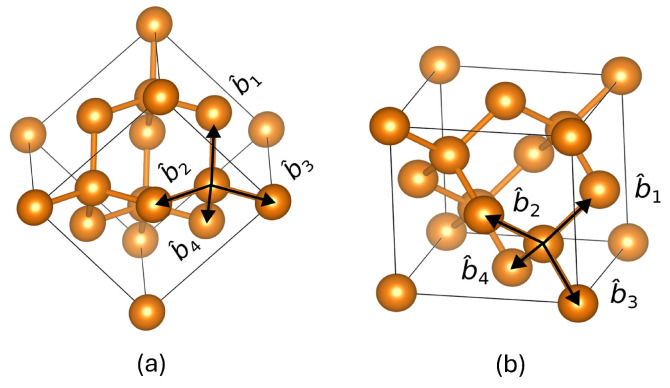
Bond vectors of the (**a**) Si(111) and (**b**) Si(001) surface.

In the silicon structures Si(001) and Si(111) [14], the bond vector directions are illustrated by black arrows, labeled ${\hat{b}}_{1},{\hat{b}}_{2},{\hat{b}}_{3}$, and ${\hat{b}}_{4}$, as depicted in Figure 3. These bond vectors can be analyzed and described in terms of Cartesian coordinates. Drawing upon previous studies, the bond orientations of the Si(111) and Si(001) structures can be represented as follows [15]

(6) $$\begin{array}{ccc} {\hat{b}}_{1} & =\left(\begin{array}{c} 0\\ 0\\ 1 \end{array}\right),\;{\hat{b}}_{2} & =\left(\begin{array}{c} \sin(\beta )\\ 0\\ \cos(\beta ) \end{array}\right),\\ {\hat{b}}_{3} & =\left(\begin{array}{c} -\sin(\beta )/2\\ \sqrt{3}\sin\left(\beta /2\right)/2\\ \cos(\beta ) \end{array}\right),\;{\hat{b}}_{4} & =\left(\begin{array}{c} -\sin\left(\beta /2\right)/\sqrt{2}\\ -\sin\left(\beta /2\right)/\sqrt{2}\\ \cos(\beta /2) \end{array}\right) \end{array}$$

(7) $$\begin{array}{ccc} {\hat{b}}_{1} & =\left(\begin{array}{c} -\sin\left(\beta /2\right)/\sqrt{2}\\ -\sin\left(\beta /2\right)/\sqrt{2}\\ -\cos(\beta /2) \end{array}\right),\;{\hat{b}}_{2} & =\left(\begin{array}{c} -\sin\left(\beta /2\right)/\sqrt{2}\\ \sin\left(\beta /2\right)/\sqrt{2}\\ -\cos(\beta /2) \end{array}\right),\\ {\hat{b}}_{3} & =\left(\begin{array}{c} -\sin\left(\beta /2\right)/\sqrt{2}\\ \sin\left(\beta /2\right)/\sqrt{2}\\ \cos(\beta /2) \end{array}\right),\;{\hat{b}}_{4} & =\left(\begin{array}{c} \sin\left(\beta /2\right)/\sqrt{2}\\ -\sin\left(\beta /2\right)/\sqrt{2}\\ \cos(\beta /2) \end{array}\right) \end{array}$$

where $\beta =109.47^{\circ }$, and the bond vectors are denoted as ${\hat{b}}_{1},{\hat{b}}_{2},{\hat{b}}_{3}$, and ${\hat{b}}_{4}$. Equations (6) and (7) provide the corresponding bond vector expressions for Si(111) and Si(001) surfaces, respectively.

Another crucial aspect is that the input field interacting with the material can be represented as

(8) $$E_{\mathrm{in}}=\left(\begin{array}{c} F_{\omega ,p}\cos\theta_{i,\omega }\sin\psi \\ F_{\omega ,s}\cos\psi \\ F_{\omega ,p}\sin\theta_{i,\omega }\sin\psi \end{array}\right)$$

where ψ corresponds to the incomming fundamental polarization angle and is $0^{\circ }$ for the *s*-polarization and $90^{\circ }$ for the *p*-polarization, while $\theta_{i,\omega }$ is the incident angle relative to the surface, and $F_{\omega ,p}$ is the Fresnel coefficient which depends on the refractive indices. To review the output field from the resulting polarization, the far-field can be applied to the polarization result according to Equation (8).

(9) $$E_{\mathrm{ff},\mathrm{int}}\left(r\right)\propto F_{2\omega }\left[I-{\hat{k}}_{\mathrm{out}}{\hat{k}}_{\mathrm{out}}\right]\cdot P_{D}^{\left(2,\mathrm{interface}\right)}$$

where I is the identity matrix and ${\hat{k}}_{\mathrm{out}}$ is the unit vector in the direction of the outgoing wave, and $P_{D}^{\left(2,\mathrm{interface}\right)}$ is the second-order dipolar polarization at the interface. The total SHG intensity of the considered MG-Si interface in this work is thus calculated as the sum of the dipolar silicon and malachite green far fields omitting quadrupole and spatial dispersion. This is because (1) the quadrupole and spatial dispersion SHG contribution in Si to the RASHG intensity profile is almost similar to those of the interface SHG dipole source [14] and (2) the main contribution of the SHG signal will come from the MG molecule if we apply their resonance frequency as the input fundamental wave following Ref. [11] hence the SHG contribution from Si substrate is small.

To enhance the SBHM model accuracy, we also investigate the adsorption geometry of MG molecule on Si(001) surface by means of density functional theory (DFT) calculations, using ultrasoft pseudopotentials as implemented in the Quantum ESPRESSO [19] package. The exchange–correlation interaction is described by the generalized gradient approximation (GGA) proposed by Perdew–Burke–Ernzerhof [20]. Wave functions and augmentation charge are expanded by a plane wave basis set with the kinetic energies of 60 and 480 Ry, respectively. Firstly, the isolated MG molecule and the Si(001) surface are fully relaxed separately. Translation and rotation transformations are applied to the MG molecule to search for the optimized structure.

## 3. Results and Discussion

Before we delve into the calculation results, we need to address several issues regarding the model validation with the existing RASHG experiments. Specifically, the facet orientation of the Si substrate in the work of Kitkeva et al. [11] is not clearly declared, whereas, in the work of Gassin et al. [10], a Si(111) facet was likely used as the MG-Si interface. Indeed, we found that the rotational anisotropy SHG (RASHG) can be accurately fitted when considering a malachite green (MG)-Si(111) surface corresponding to a C${‌}_{2v}$/C${‌}_{3v}$. Additionally, following the findings of Kitkeva et al., the contribution from the Si facet is significantly smaller, by a factor of hundreds, due to the choice regarding the incoming wavelength, which aligns with the second-harmonic generation (SHG) resonance frequency of MG. As a result, the SHG signal is predominantly influenced by the MG contaminant. We therefore incorporate this information into our model by addressing a higher weight factor for the MG SHG field. If this assumption is taken into account, it does not make a significant difference whether we model the surface as Si(111) or Si(001) as the dominant SHG intensity arises from MG, which has a C${‌}_{2v}$ point group symmetry.

### 3.1. SBHM Simulation for MG/Si(111)

In this section, we will apply the SBHM to reproduce the RASHG experimental result in Ref. [11] for an MG/Si(111) interface as a validation. We will compare the SHG nonlinear tensor and explain our results. Afterwards, we will apply the validated model supported by DFT calculation to predict the RASHG experiments for an MG/Si(001) interface. Here, we begin by analyzing the susceptibility tensor of the Si(111) substrate and the MG contaminants before comparing with the group theory literature.

#### 3.1.1. Susceptibility Tensor of Si(111)

As shown in Figure 3a, the Si(111) surface structure displays $C_{3v}$ symmetry. According to the study by Alejo-Molina et al. (2014) [15], the susceptibility tensor for the Si(111) surface with $C_{3v}$ symmetry is defined as follows:

(10) $$\chi_{\mathrm{Si}(111)}=\left(\begin{array}{c} \left(\begin{array}{ccc} 0 & -2d_{222} & d_{131}\\ -2d_{222} & 0 & 0\\ d_{131} & 0 & 0 \end{array}\right)\\ \left(\begin{array}{ccc} -d_{222} & 0 & 0\\ 0 & d_{222} & d_{131}\\ 0 & d_{131} & 0 \end{array}\right)\\ \left(\begin{array}{ccc} d_{311} & 0 & 0\\ 0 & d_{311} & 0\\ 0 & 0 & d_{333} \end{array}\right) \end{array}\right)$$

employing the SBHM method, the bond vector calculations for the Si(111) structure yield a susceptibility tensor as presented in Equation (11) [7,15]:

(11) $$\chi_{\mathrm{Si}(111)}=\left(\begin{array}{c} \left(\begin{array}{ccc} 0 & -\frac{3\alpha_{l}}{4}\sin^{3}\beta & \frac{3\alpha_{l}}{2}\cos\beta \sin^{2}\beta \\ -\frac{3\alpha_{l}}{4}\sin^{3}\beta & 0 & 0\\ \frac{3\alpha_{l}}{2}\cos\beta \sin^{2}\beta & 0 & 0 \end{array}\right)\\ \left(\begin{array}{ccc} -\frac{3\alpha_{l}}{4}\sin^{3}\beta & 0 & 0\\ 0 & \frac{3\alpha_{l}}{4}\sin^{3}\beta & \frac{3\alpha_{l}}{2}\cos\beta \sin^{2}\beta \\ 0 & \frac{3\alpha_{l}}{2}\cos\beta \sin^{2}\beta & 0 \end{array}\right)\\ \left(\begin{array}{ccc} \frac{3\alpha_{l}}{2}\cos\beta \sin^{2}\beta & 0 & 0\\ 0 & \frac{3\alpha_{l}}{2}\cos\beta \sin^{2}\beta & 0\\ 0 & 0 & \alpha_{u}+3\alpha_{l}\cos^{3}\beta \end{array}\right) \end{array}\right)$$

where $\alpha_{u}$ and $\alpha_{l}$ are the hyperpolarizability of upper and lower silicon bond vectors. Equation (10) outlines a group theory approach that identifies the non-zero tensor components, specifically $d_{131}=d_{311}$, $d_{222}$, and $d_{333}$, as reported by Alejo-Molina et al. (2014) [15]. This method is further explored within the SBHM framework from a group theory perspective [21].

(12) $$d_{131}=d_{311}\to \frac{3\alpha_{l}}{2}\cos\beta \sin^{2}\beta ,\;d_{222}\to \frac{3\alpha_{l}}{8}\sin^{3}\beta ,\;$$

and

(13) $$\;d_{333}\to \alpha_{u}+3\alpha_{l}\cos^{3}\beta .$$

#### 3.1.2. Susceptibility Tensor of Malachite Green (MG)

Based on the study of Eckenrode et al. (2020) [23], the malachite green (MG) structure exhibits $C_{2v}$ symmetry. Therefore, the susceptibility tensor for malachite green is represented in Equation (14). Additionally, the susceptibility tensor derived from the SBHM method, based on the bond vectors in Equation (17), takes the form presented in Equation (15).

(14) $$\chi_{\mathrm{MG}}=\left(\begin{array}{c} \left(\begin{array}{ccc} 0 & 0 & d_{131}\\ 0 & 0 & 0\\ d_{131} & 0 & 0 \end{array}\right)\\ \left(\begin{array}{ccc} 0 & 0 & 0\\ 0 & 0 & d_{232}\\ 0 & d_{232} & 0 \end{array}\right)\\ \left(\begin{array}{ccc} d_{311} & 0 & 0\\ 0 & d_{322} & 0\\ 0 & 0 & d_{333} \end{array}\right) \end{array}\right).$$

(15) $$\chi_{MG}=\alpha_{MG}\left(\begin{array}{c} \left(\begin{array}{ccc} 0 & 0 & -2B\\ 0 & 0 & 0\\ -2B & 0 & 0 \end{array}\right)\\ \left(\begin{array}{ccc} 0 & 0 & 0\\ 0 & 0 & 0\\ 0 & 0 & 0 \end{array}\right)\\ \left(\begin{array}{ccc} -2B & 0 & 0\\ 0 & 0 & 0\\ 0 & 0 & 1-2\sin^{3}\left(\gamma \right) \end{array}\right) \end{array}\right)$$

where $B=\cos^{2}\left(\gamma \right)\sin\left(\gamma \right)$. Equation (13), which was obtained from group theory and the tensor in Equation (14), matches the symmetry of the malachite green molecule, namely $C_{2v}$.

(16) $$d_{131}=d_{311}\to -2B\alpha_{MG},\;d_{333}\to \alpha_{MG}\left(1-2\sin^{3}\gamma \right)\;$$

as described in Refs. [10,11], the quantities $d_{333}$, $d_{311}$, and $d_{113}$ are the three non-vanishing and independent quadratic susceptibility tensor components associated with an isotropic and achiral interface, and the subscripts represent the cartesian coordinates in the laboratory frame.

#### 3.1.3. SHG Total Intensity MG/Si(111)

**Figure 4:**
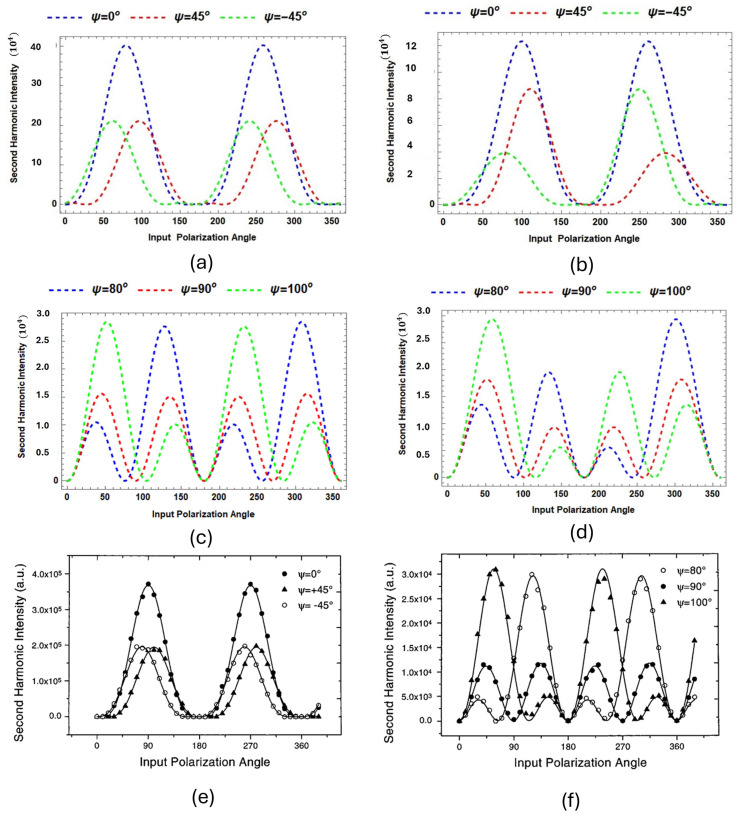
Total simulated (**a**–**d**) and experimental (**e**,**f**) RASHG intensity, where in (**a**,**c**) the normal *z*-axis of the Si substrate and the vertical MG ${\hat{b}}_{5}$ bond vector are parallel ($theta=0^{\circ }$), whereas in (**b**,**d**) the tilting angle between the normal *z*-axis and ${\hat{b}}_{5}$ is $\theta =8^{\circ }$. The polarization angle is evaluated for ψ=$0^{\circ }$, $-45^{\circ }$, and $45^{\circ }$ (left side), while $\psi =80^{\circ }$, $90^{\circ }$, and $100^{\circ }$ (right side). The RASHG experiments in (**e**,**f**) were reprinted/adapted with permission from Ref. [11]. 2000. American Chemical Society.

**Figure 5:**
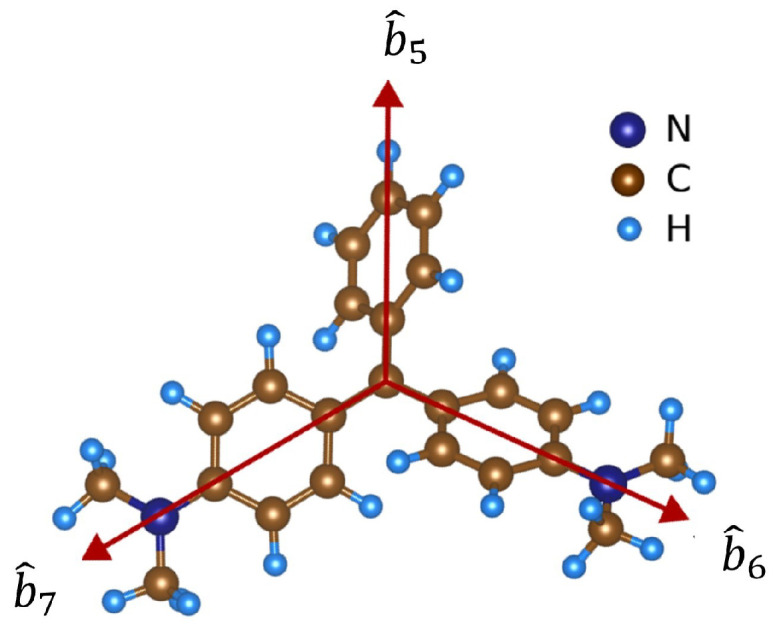
Atomic structure of isolated malachite green. Red arrows correspond to the vector direction of each branch of the molecule, i.e., ${\hat{b}}_{5}$, ${\hat{b}}_{6}$, and ${\hat{b}}_{7}$. Navy, gray, and cyan spheres represent the nitrogen, carbon, and hydrogen atoms, respectively. The structural models are visualized by using VESTA [24].

The SHG intensity obtained from the simulation is a consequence of the polarization occurring within both structures, silicon and malachite green (MG). The resulting second-harmonic generation (SHG) intensity pattern is presented in Figure 4. As noted by Kikteva et al. [11], one of the angles between vector ${\hat{b}}_{5}$ (see Figure 5) and the *z*-axis is θ=$8^{\circ }$. This leads to an asymmetrical SHG intensity pattern from SBHM simulation, differing from the experimental results shown in Figure 4c,f.

Conversely, when vector ${\hat{b}}_{5}$ is parallel to the *z*-axis θ=$0^{\circ }$, the SHG intensity pattern from the symmetric SBHM simulation is consistent with the experimental observations for all the polarization angles. The variation in intensity patterns observed between the experimental results and the SBHM simulation at the bond vector angle ${\hat{b}}_{5}$ relative to the *z*-axis ($\theta =8^{\circ }$) may be due to differences in the coordinate selection of the incoming and outgoing angle as well as the positioning of the SHG detector with respect to the normal *z*-axis coordinate, which is not clearly explained. Therefore, the symmetrical SHG intensity pattern detected in the experimental data strongly suggests two possibilities: (1) the MG orientation is oriented vertically normal to the Si substrate so that the bond vector ${\hat{b}}_{5}$ of MG is aligned parallel to the *z*-axis, or (2) the MG orientation is not vertically normal to the Si substrate but has a certain tilting angle (e.g., 8 degrees, as claimed by Kitkeva et al.). However the symmetrical SHG intensity signal implies that the SHG detector is aligned in such a way so that the normal z-line in the optical plane is parallel to the the tilting angle of the MG pollutant whose bond vector is ${\hat{b}}_{5}$. We will show in the latter subsection that DFT suggests that the second possibility is more likely.

### 3.2. SBHM-DFT Prediction for MG/Si(001)

**Figure 6:**
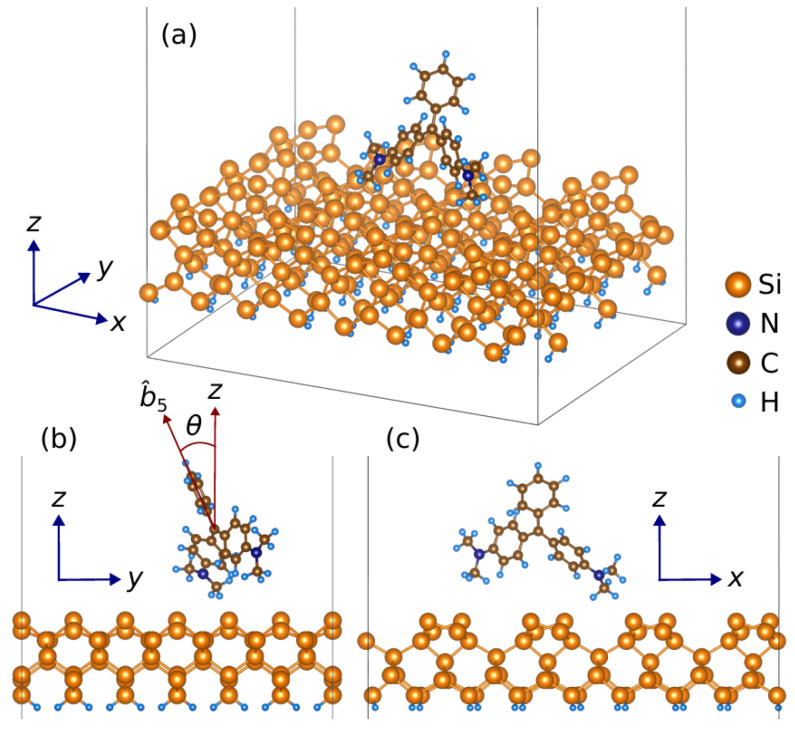
(**a**) Optimized geometrical structure of MG/Si(001) obtained by DFT calculations. 2D Sideview of the optimal orientation when viewed from the (**b**) z-y and (**c**) (z,x) plane cut.

**Figure 7:**
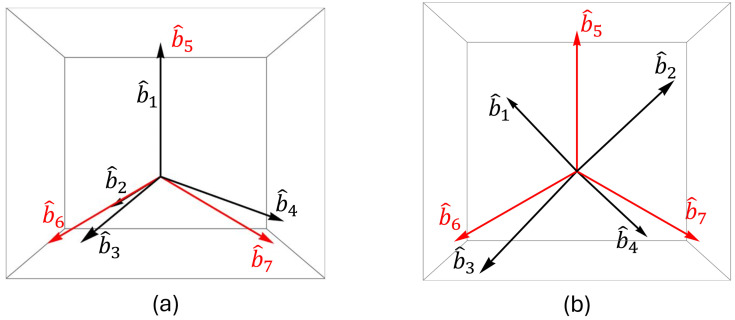
Bond vectors of the (**a**) MG/Si(111). (**b**) MG/Si(001) surface. The red bond vectors belong to MG and the black bond vectors belong to Si. No tilting between MG and Si is initially assumed.

To obtain the correct molecular orientation and bond vector for the MG-Si(001) surface, we apply DFT as described in the Methods section to support the SBHM model. The molecular structure of malachite green (MG) is depicted in Figure 5, where its non-chiral effective bond vectors are indicated by red arrows and labeled as ${\hat{b}}_{5}$, ${\hat{b}}_{6}$, and ${\hat{b}}_{7}$. In Figure 5, bond vectors for MG are presented with a shape like an inverted Y letter, with each bond vector ${\hat{b}}_{5}$, ${\hat{b}}_{6}$, and ${\hat{b}}_{7}$ described as follows:

(17) $$\begin{array}{cccc} {\hat{b}}_{5} & =\left(\begin{array}{c} 0\\ 0\\ 1 \end{array}\right),\;{\hat{b}}_{6} & =\left(\begin{array}{c} \cos(\gamma )\\ 0\\ -\sin(\gamma ) \end{array}\right),\;{\hat{b}}_{7} & =\left(\begin{array}{c} -\cos(\gamma )\\ 0\\ -\sin(\gamma ) \end{array}\right) \end{array}$$

here, $\gamma =30^{\circ }$ represents the angle between bond vectors $b_{6}$ and $b_{7}$ relative to the *x*-axis. The optimized structure of the MG-Si(001) molecule is shown in Figure 6. When malachite green (MG) is attached to the silicon substrate, Figure 6 presents an illustration depicting an arbitrary orientation of MG on the surface of the silicon substrate, with the θ representing the angle between the bond vector ${\hat{b}}_{5}$ of MG and the *z*-axis. Furthermore, the bond vector orientation of the MG/Si surface for the SBHM is presented in Figure 7.

Following the analysis of each bond vector, a rotation matrix is applied to rotate them around the *z*-axis, as described in Equation (5). The subsequent step involves the formulation of second-order polarization, specifically second-harmonic generation, in accordance with Equation (3).

To model the Si(001) surface, we use a five-layer slab and introduce dihydrogen to terminate the dangling bond of the Si atoms at the bottom side of the surface (see Figure 6). After relaxation, the Si atoms at the top side of the surface form dimers, as clearly shown in Figure 6c. To sufficiently accommodate the MG molecule adsorption, a 4×3 large supercell of Si(001) (30.94×23.20 Å${‌}^{2}$) containing 336 atoms is constructed. A vacuum layer is set to approximately 34 Å to avoid the unphysical long-range interactions due to the lattice periodicity. As the cell is large enough, the irreducible Brillouin zone (BZ) is only sampled at Γ-point. Instead of fully relaxing the adsorbed system, which is computationally costly, we manually place the MG molecule on top of the Si(001) surface in various positions and check the total energy.

#### 3.2.1. Predicted RASHG Total Intensity MG/Si(001) from SBHM

**Figure 8:**
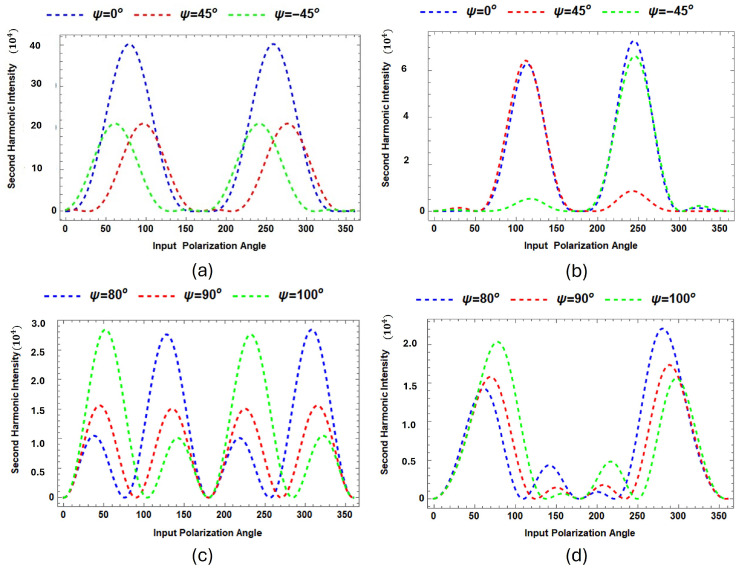
Total predicted RASHG intensity for MG-Si(001) at polarization input angles ψ=$0^{\circ }$, $-45^{\circ }$, and $45^{\circ }$ (left side), while $\psi =80^{\circ }$, $90^{\circ }$, and $100^{\circ }$ (right side). For (**a**,**c**), no tilting angle is applied ($\theta =0^{\circ }$), whereas for (**b**,**d**) a tilting angle of $\theta =8^{\circ }$ is applied.

Figure 8 presents the predicted RASHG (Rotational Anisotropic Second Harmonic Generation) simulation plots comparing the second harmonic intensities for MG-Si(001) at different polarization input angles using the SBHM. The plots explore how these angles, combined with the presence or absence of a tilting angle, affect the total predicted RASHG intensity. In Figure 8a,b, the input polarization angles are $\psi =0^{\circ }$, $45^{\circ }$, and $-45^{\circ }$, while in Figure 8c,d, the input angles are $\psi =80^{\circ }$, $90^{\circ }$, and $100^{\circ }$. For Figure 8a,c, no tilting angle is applied ($\theta =0^{\circ }$), while Figure 8b,d incorporate a tilting angle of $\theta =8^{\circ }$.

In Figure 8a, where no tilting is assumed, the SBHM simulation produces the strongest SHG intensity with prominent peaks near $45^{\circ }$ and $225^{\circ }$, while $\psi =45^{\circ }$ and $-45^{\circ }$ show lower SHG peak intensities. In contrast, Figure 8b shows that applying a tilting angle breaks the symmetry and reduces the intensity and slightly shifts the peaks. For Figure 8c,d, with $\psi =80^{\circ }$, $90^{\circ }$, and $100^{\circ }$, the no-tilt condition (Figure 8c) results in more spread-out and less symmetric peaks compared to Figure 8a. The tilting angle in Figure 8d further breaks the symmetry of Figure 8c, particularly for $\psi =80^{\circ }$ and $\psi =100^{\circ }$, with phase shifts becoming apparent. Overall, the absence of a tilting angle generally result in a symmetric SHG intensity, whereas the introduction of tilting breaks the symmetry. This suggests that the MG surface orientation play significant roles in second harmonic generation RASHG profile and one can use SBHM to determine the tilting angle. Furthermore, the RASHG simulation also show that when the resonance wavelength for MG is selected in the simulation the dominance of MG SHG contribution compared to the silicon substrate at the surface is very significant, roughly by the order of hundreds thus making it very suitable to detect organic contaminants. We invite the experimenter to validate our simulated MG-Si(001) RASHG profile.

#### 3.2.2. MG/Si(001) Density of States and Optimal Surface Orientation from DFT

**Figure 9:**
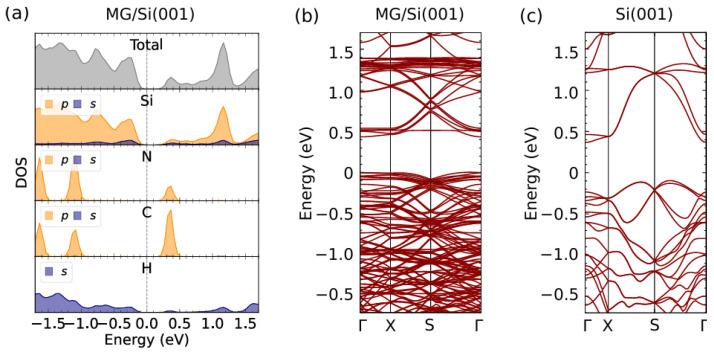
Density of states (**a**) and band structures (**b**) of MG/Si(001). The dominant contribution comes from the *p*-orbital of Si. Meanwhile, the *p*-orbital of carbon atom from MG creates acceptor states at the conduction band. The bandgap is about ∼0.3 eV. (**c**) The band structure of Si(001) without MG.

In Figure 9, we show the density of states of Si(001)+MG at a relaxed geometry obtained from the density functional theory calculation. We obtained the calculated bandgap to be about 0.3 eV, which is much lower than the bulk gap ∼1.1 eV. The previous work using a GGA functional obtained the same bandgap regarding ∼0.3 eV, while using a hybrid functional can reach a direct band gap of ∼1.1 eV and the global (indirect) gap ∼0.6 eV [25]. The angle-resolved photoemission spectroscopy on the Si-surface shows that the valence band is located around 0.75 eV below the Fermi level [26]. We note that the presence of MG provides acceptor states above the Fermi level. The band structure is sensitive upon a slight variation in the θ angle (see Figure 5). The main contribution of the orbitals around the Fermi level comes from the $sp^{3}$ hybridization of the Si and *p* orbitals of C from MG.

**Figure 10:**
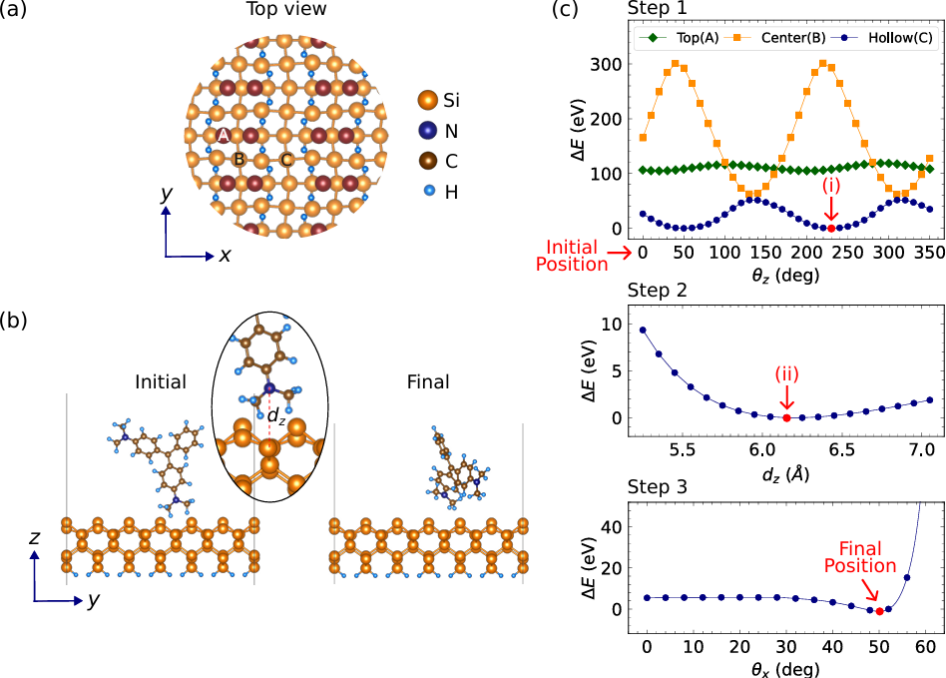
(**a**) Top view of the Si(001) surface. Red spheres represent the Si atoms in the top layer. Top, center, and hollow sites are assigned to A, B, and C, respectively. (**b**) Side view of the initial and final structures of MG/Si(001) system. (**c**) Evolution of the MG molecule orientation from its initial to final position as they seek the lowest energy, ΔE where $d_{z}$ corresponds to the distance between N and Si atoms at the hollow site whereas $\theta_{z}$ and $\theta_{x}$ refer to the tilting angle with respect to the *z* and *x* coordinate.

Meanwhile, in Figure 10 we present a detailed DFT analysis of the MG/Si(001) optimal molecular orientation which is important when modelling the bond vector using SBHM. In Figure 10a, a top view of the Si(001) surface is provided, with the silicon atoms in the top layer represented by red spheres. The figure highlights three specific adsorption sites: A (top), B (center), and C (hollow). These sites are crucial for understanding the interactions between the molecule and the surface. The atoms involved are color-coded, with orange for silicon (Si), blue for nitrogen (N), gray for carbon (C), and white for hydrogen (H), providing a clear representation of the molecular system. In Figure 10b, the side view depicts the initial and final configurations of the molecule on the Si(001) surface. The molecule moves from its initial position to the final position at the hollow site, where the distance between the nitrogen and silicon atoms ($d_{z}$) plays a significant role in the adsorption process and is shown in Figure 10c.

We also depict the energy behavior over three steps. Step 1 shows the energy variation as a function of the rotational angle ($\theta_{z}$) for the three adsorption sites, with the hollow site (C) yielding the lowest energy, making it the most favorable adsorption site. Step 2 focuses on the relationship between the distance ($d_{z}$) and the adsorption energy, demonstrating that as the molecule approaches the surface, the energy decreases, reaching a minimum near 6.7 Å, indicating optimal adsorption distance. Step 3 further refines the rotational angle, showing minimal energy at specific orientations, signifying the system’s most stable configuration which is at a tilting angle around 50${‌}^{\circ }$. RASHG experiments combined with SBHM can validate our DFT calculation in the future.

### 3.3. Simulated Photonic Crystal Cavity Design

**Figure 11:**
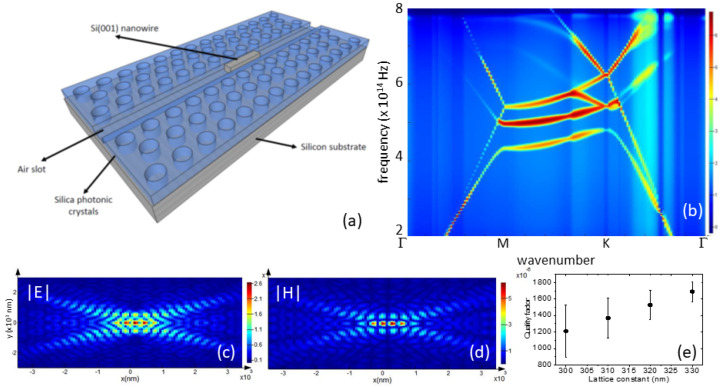
Photonic crystal device (PCD) based on Si(001) nanowire. (**a**) Design of SiO${‌}_{2}$ PCD with Si(001) nanowire inside the air slot. (**b**) Photonic bandstructure calculation for photonic crystals with a lattice constant of 320 nm and a hole radius of 109 nm. (**c**) Calculated electric |E| and (**d**) magnetic |H| field distributions. (**e**) Quality factors of nanowire cavities as a function of lattice constants. Other parameters are mentioned in the text.

Since the SHG intensity is very small compared to the linear optics, we propose a method to enhance the RASHG signal through a photonic crystal cavity (PhC) [27]. In Figure 11a, we present a Si nanowire cavity inside an air-slot SiO${‌}_{2}$ PhC design, as demonstrated previously for an ultrahigh *Q* cavity [28]. We used finite-difference time-domain commercial software Lumerical(2024R1) to simulate the electromagnetic fields inside the PhC. Further, we defined the frequency as 4.9 × 10${‌}^{14}$ Hz, which closely matches the frequency of RASHG Si[001] [14]. For such a purpose, we defined the hole radius of 109 nm, while the lattice constants, *a*, vary between 300 and 330 nm. The air slot in the PhC [28] has parameters of width and height of 0.25a, and the total size of the calculation is 23a×$12a\sqrt{2}$. For the nanowire, it has a length and side length of 2 and 0.17*a*, respectively. Figure 11b shows the calculated photonic bandstructure calculation with the bandgap between 4.4 and 5.4 × 10${‌}^{14}$ Hz. We found that the electric and magnetic fields are strongly confined in the Si nanowire, as shown in Figure 11c,d, respectively. The mode volume for the PhC can be estimated to be 0.18${\left(\lambda /n\right)}^{3}$. For quality factors, we obtained the values between 1200 and 1800 and the highest value determined for *a* equal to 330 nm, as shown in Figure 11e. 

Those quality factors are still smaller than those obtained for the nanowire in Si PhC [28] due to a much lower effective refractive index (closer to SiO${‌}_{2}$). However, from the value of the Purcell factor [28], which is about 4000, we expected that we can increase the RASHG signal to a similar value. The mode orientation that is along the nanowire side length matches well with the RASHG polarizations [14], and therefore a simulated PCD is the perfect solution for the enhancement of sensitivity from this RASHG-based contaminant sensor.

### 3.4. Future Work in Pollutant Detection Using NLO

Finally, as a future direction of this work, in addition to malachite green (MG), several other low-symmetry pollutants can be explored using the SBHM model, provided they possess covalent or hydrogenic bonds and exhibit point group symmetries distinct from the substrate. Crystal violet (CV), belonging to the D${‌}_{3h}$ point group, and Rhodamine B, categorized under the C${‌}_{2v}$ point group, are synthetic dyes commonly studied for their environmental impact. Phenolic compounds like chlorophenols, specifically in the C${‌}_{2v}$ point group for mono-chlorophenols, are widely used in industrial applications but pose significant health risks. Bisphenol A (BPA), with a C${‌}_{2}$ symmetry, is a major concern due to its endocrine-disrupting properties. Similarly, polychlorinated biphenyls (PCBs), classified under the C${‌}_{2v}$ point group for mono-substituted variants, are known environmental pollutants. Pesticides such as DDT, characterized by the C${‌}_{1}$ point group, and Atrazine, which falls under the C${‌}_{3v}$ point group, continue to be debated for their ecological and health impacts. Polycyclic aromatic hydrocarbons (PAHs), with D${‌}_{2h}$ symmetry, and nitroaromatic compounds like 2,4-Dinitrotoluene, classified under C${‌}_{1}$ symmetry, are notable for their persistence in the environment and potential carcinogenicity. Each of these compounds, from industrial dyes to pesticides and hydrocarbons, share molecular structures with low symmetry, making them ideal candidates for surface-sensitive techniques like second-harmonic generation (SHG), and they can be analyzed using SBHM. The primary requirement for the SBHM model’s application is that these molecules exhibit distinct point group symmetries that differ from those of the substrate. For instance, MG, which has C${‌}_{2v}$ symmetry at the surface, contrasts with the Si(111) substrate, which possesses C${‌}_{3v}$ symmetry at the surface and Th symmetry in the bulk. This difference in the symmetry between the pollutant and the substrate allows the SBHM to effectively detect changes in the molecular orientation at the surface, providing valuable insights into pollutant–surface interactions.

## 4. Conclusions

Based on the simulation results that have been carried out in this study, investigations on the surface of silicon (001) with MG regarding both experimental and simulation results obtained the same intensity pattern for all the polarization angles. This result shows that there is a polarization contribution that occurs on silicon and MG on the surface. Then, the structure of MG by reviewing the center like an inverted Y can be used as a reference for further research. Finally, with nanophotonics, a simulated PCD was proposed to enhance the RASHG signal for improving the sensitivity of Si(001) to detect MG.

## Data Availability

The data concerning all the results in this work are not publicly available at this moment but may be obtained from the authors upon request.
